# Characterization of dermatofibromas and epidermoid cysts using dermoscopy-guided high-frequency ultrasound imaging

**DOI:** 10.1007/s40477-026-01131-1

**Published:** 2026-03-27

**Authors:** P. Hamilton-Meikle, M. Boostani, N. Kiss, G. Pellacani, P. Holló, N. Wikonkál, S. Bozsányi, A. Bánvölgy, C. Cantisani

**Affiliations:** 1 Department of Dermatology, Dermatooncology and Venerology, Semmelweis University, Hungaria, Hungary; 2 Department of Dermatology, Roswell Park Comprehensive Cancer Center, Buffalo, NY USA; 3 UOC of Dermatology, Department of Clinical and Cardiovascular Sciences, Sapienza University of Rome, Rome, Italy; 4 Military Hospital, North-Pest Central Hospital, Hungaria, Hungary; 5 UOC of Dermatology, Department of Medical and Cardiovascular Science, Sapienza Medical School of Rome, Viale Del Policlinico 155 161, Rome, Italy

**Keywords:** Dermatofibroma, Epidermoid cyst, Dermoscopy-guided high-frequency ultrasound, Differential diagnosis

## Abstract

**Purpose:**

Epidermoid cysts and dermatofibromas may resemble malignant lesions, requiring accurate non-invasive differentiation. Our objective was to assess dermoscopy-guided high-frequency ultrasound (DG-HFUS) in distinguishing these lesions.

**Methods:**

Nine lesions (four epidermoid cysts, five dermatofibromas) were examined using DG-HFUS with a 33 MHz linear probe in B-mode. The dermoscopic modality guided probe placement. For each lesion, a minimum of 10 consecutive frames were acquired to ensure measurement reproducibility. Lesions were evaluated based on predetermined characteristics.

**Results:**

Epidermoid cysts presented as well-circumscribed, oval lesions located in the dermis or dermal–subcutaneous border, with depths of 1.4–8.7 mm. All showed mixed echogenicity with hyperechoic foci and bands. Dermatofibromas were smaller (2.4–3.5 mm), within the dermis, hypoechoic with scattered hyperechoic areas, poorly defined margins, and 3–5 hypoechoic bands extending into surrounding structures.

**Conclusion:**

DG-HFUS reliably correlated dermoscopic and sonographic features, enhancing non-invasive diagnosis of benign cutaneous lesions.

## Case report

Cutaneous nodules are common in clinical practice and are most often benign, though they may occasionally mimic malignant lesions. Epidermoid cysts and dermatofibromas are frequently encountered benign lesions with distinct origins and management strategies. Epidermoid cysts are encapsulated subepidermal nodules filled with laminated keratin, arising from the follicular infundibulum or traumatic implantation of epidermal cells [1, 2]. Clinically, they present as slow-growing, firm nodules, often with a central punctum, and may become inflamed or rupture [3]. Dermatofibromas are common dermal lesions composed of spindle-shaped fibroblasts and histiocytes, typically presenting as small, firm nodules on the extremities and usually managed conservatively [4, 5]. Despite their benign nature, both lesions may resemble other cutaneous tumors. While histopathology remains the diagnostic gold standard, clinical examination and dermoscopy offer limited information on lesion depth and internal structure, highlighting the value of high-frequency ultrasound as a non-invasive diagnostic tool.

HFUS has emerged as a valuable non-invasive imaging modality in dermatology [6–12]. Using sound waves above 20 megahertz (MHz), HFUS enables visualization of skin and subcutaneous structures without the need for invasive procedures [13]. In our study, imaging was performed at the UOC of Dermatology, Sapienza University of Rome, Italy using a compact dermoscopy-guided high frequency ultrasound (DG-HFUS) system (Dermus SkinScanner, Dermus Ltd, Budapest, Hungary), which integrates both ultrasound and dermoscopic imaging technologies [9, 11, 14, 15]. The device operates at a nominal frequency of 33 MHz (20–40 MHz range) and uses a silicone-protected scanning interface with coupling gel. An integrated optical module enables accurate lesion localization, while ultrasound images are displayed in color scale to enhance tissue contrast. The optical field of view is 15 × 15 mm, and the ultrasound scan covers up to 12 mm laterally with a penetration depth of 10 mm. Spatial resolution is 24 μm axially and 75 μm laterally. Additional scans were performed for larger lesions [16]. All images were archived on a cloud-based platform with patient demographics, diagnosis, and anatomical site.

**Fig. 1:**
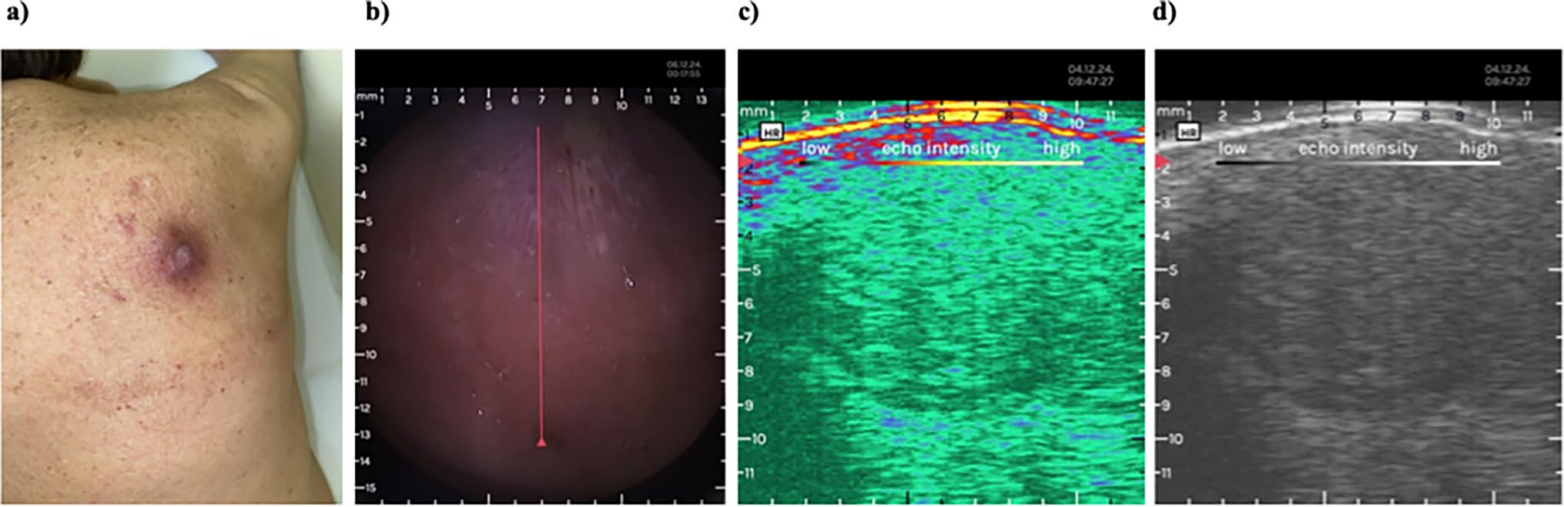
Epidermoid cyst in a 68-year-old male patient, Fitzpatrick skin type II. **a** Clinical image showing a round, purple-coloured, raised lesion with irregular shape on the left scapular region. The lesion was firm on palpation and moved with the skin. **b** Dermoscopic image of the lesion. **c** Dermoscopy-guided high-frequency ultrasound (DG-HFUS) image demonstrating an oval-shaped lesion located at the dermal–subcutaneous border, with a measured depth of 8.7 mm. **d** DG-HFUS image showing a heterogeneously hyperechoic lesion with regular margins and posterior acoustic enhancement, consistent with an epidermoid cyst. The inhomogeneous hyperechogenicity corresponds to the high keratin content characteristic of epidermoid cysts

**Fig. 2:**
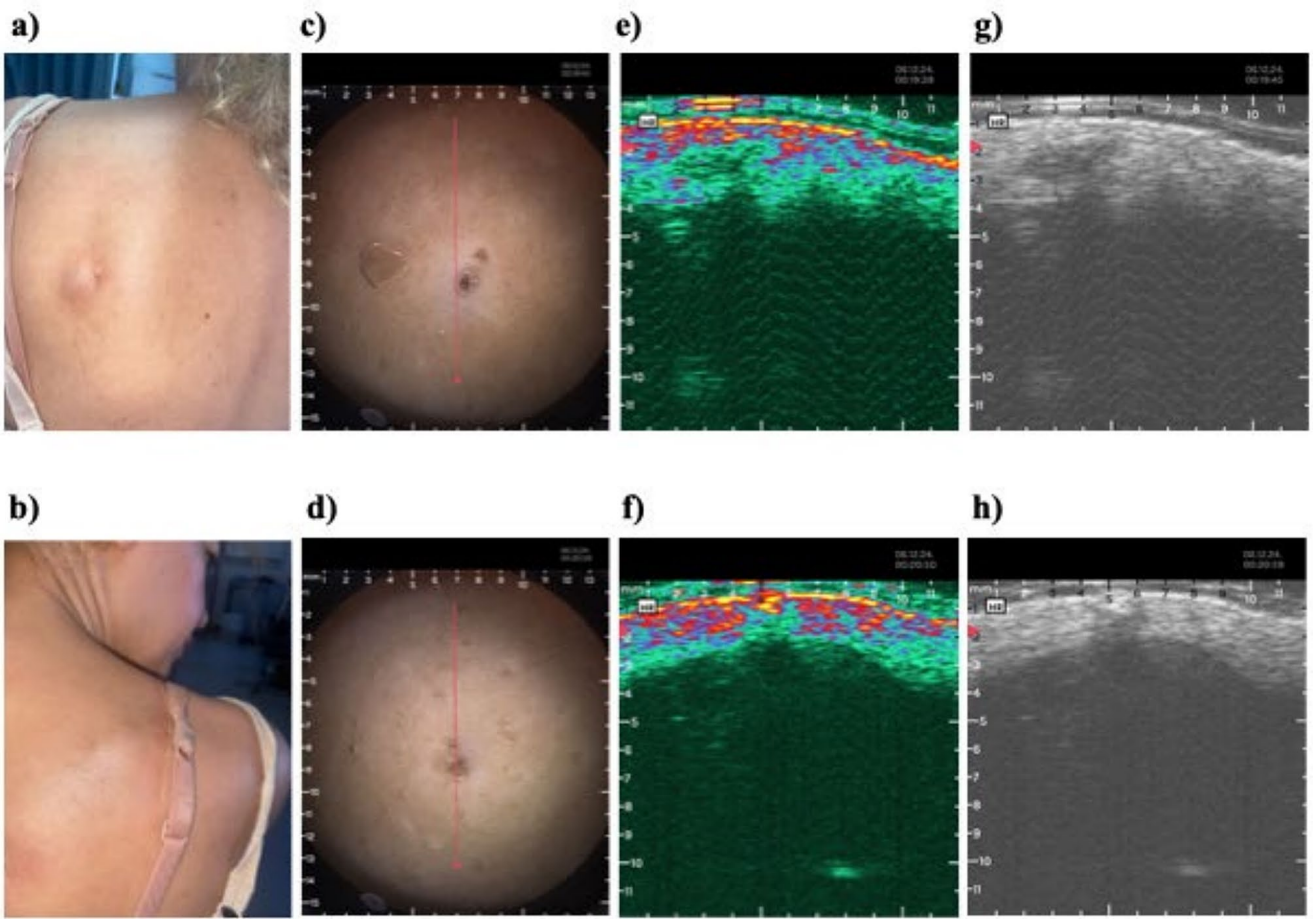
Epidermoid cysts in a 55-year-old female patient, Fitzpatrick skin type II. **a**, **b** Clinical images showing skin-coloured, round, regularly shaped, raised lesions on the left scapular region, slightly lighter than the surrounding skin, with a small central punctum. **c**, **d** Dermoscopic image of the lesions. **e**, **g** Dermoscopy-guided high-frequency ultrasound (DG-HFUS) images of lesion 1, demonstrating a dermal lesion with a depth of 2.1 mm. **f**, **h** DG-HFUS images of lesion 2, showing a dermal lesion with a depth of 1.4 mm. Both lesions appear less hyperechoic than the surrounding dermis, with focal hyperechoic areas and irregular, star-like protrusions extending into the adjacent tissue, likely corresponding to collagen bundles linking the cysts to neighbouring dermal structures. In both cases the submarine sign can be observed

**Fig. 3:**
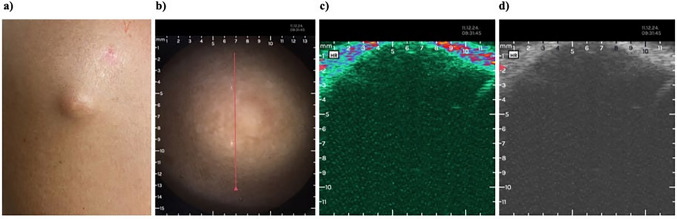
Epidermoid cyst in a 56-year-old female patient, Fitzpatrick skin type II. **a** Clinical image showing an oval-shaped, regular, raised, skin-coloured lesion located on the right deltoid region. **b** Dermoscopic image of the lesion. **c**, **d** Dermoscopy-guided high-frequency ultrasound (DG-HFUS) images demonstrating a dermal lesion with a measured depth of 2.8 mm. The lesion appears oval, well-defined, and sharply demarcated lesion that is predominantly hypoechoic with small internal hyperechoic areas and bands, consistent with keratin fibres

In our study, a total of four epidermoid cysts were examined across three patients. Clinically, the lesions were round, raised, skin-coloured in three cases, and purple coloured in one case. Under the dermoscopy imaging of the DG-HFUS device, two lesions showed a central punctum (Fig. 2.). Upon DG-HFUS examination, epidermoid cysts were typically well circumscribed and slightly irregular but with defined lateral margins, located within the dermis or at the dermal–subcutaneous border. The measured depths ranged from 1.4 mm to 8.7 mm. They demonstrated mixed echogenicity, predominantly hypoechoic with many focal hyperechoic areas or bands corresponding to keratinous material. One lesion presented as heterogenously hyperechoic due to dense keratin content and showed posterior acoustic enhancement (Fig. 1). In three cases, irregular hyperechoic protrusions extended into neighbouring tissue, resembling collagen bundles linking the lesion to surrounding skin. In two cases, the “submarine sign” (a focal, hypoechoic projection from the cyst that extends toward the epidermis) was noted (Fig. 2), in other cases, this was not present (Fig. 3).

These findings align with established sonographic features of epidermoid cysts, which typically appear as well-circumscribed, round to oval lesions within the dermis or subcutaneous tissue. They usually exhibit a cystic pattern with mixed echogenicity due to internal keratinous debris, often seen as echogenic foci or bands. The characteristic “submarine sign” may be present, representing a tract to the epidermis, and posterior acoustic enhancement is frequently observed. [3, 17].

**Fig. 4:**
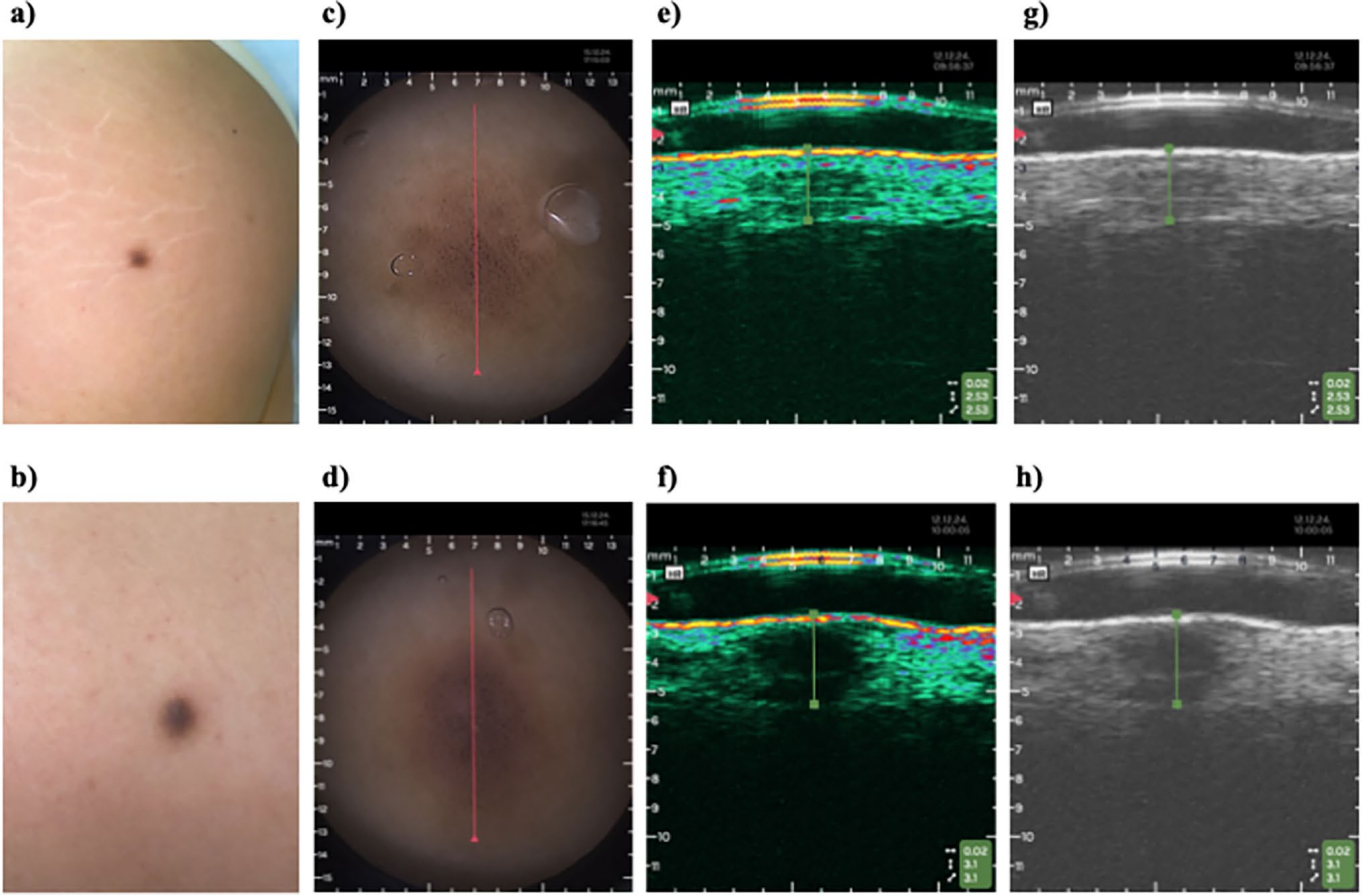
Dermatofibromas in a 23-year-old female patient, Fitzpatrick skin type II. **a**, **b** Clinical images showing flat, round, pigmented lesions with irregular borders and a darker, hyperkeratotic central area on the left thigh (**a**) and right thigh (**e**). **c**, **d** Dermoscopic images of the lesions. **e**, **g** Dermoscopy-guided high-frequency ultrasound (DG-HFUS) images of lesion 1, demonstrating a dermal lesion with a depth of 2.4 mm. **f**, **h** DG-HFUS image of lesion 2, showing a dermal lesion with a depth of 3.0 mm. Both lesions appear predominantly hypoechoic with small internal hyperechoic areas, show poorly defined margins, and are not sharply demarcated from the surrounding dermal structures. These sonographic features are consistent with dermatofibroma

**Fig. 5:**
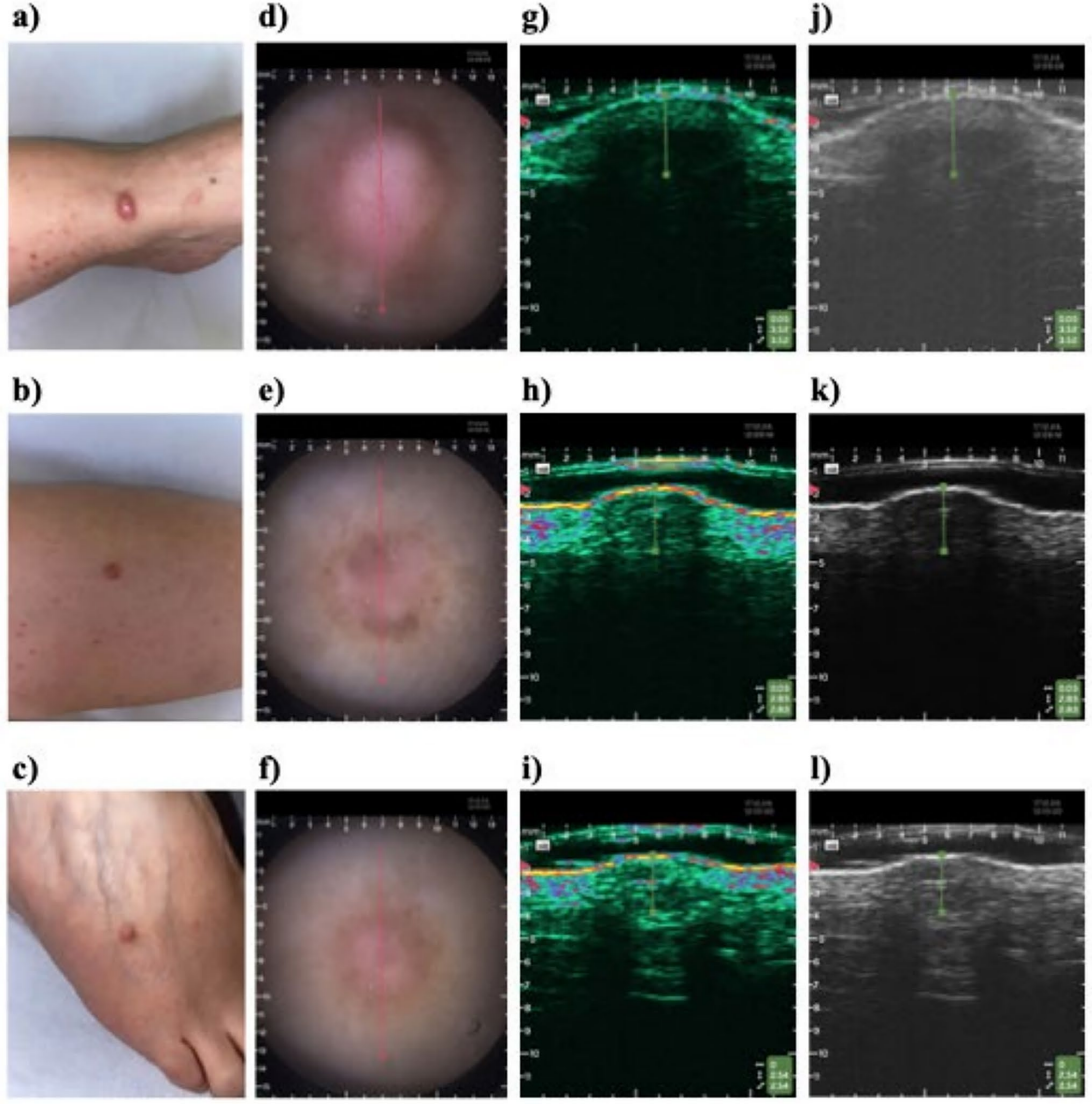
Dermatofibromas in a 70-year-old female patient, Fitzpatrick skin type II. **a**–**c** Clinical images showing oval (**a**) or round (**b**, **c**) pigmented, raised lesions on the lower extremity. **d**–**f** Dermoscopic image showing a skin-coloured central area with irregular peripheral pigmentation composed of brown and red structures. **g**–**l** Dermoscopy-guided high-frequency ultrasound (DG-HFUS) images demonstrating predominantly hypoechoic dermal lesions with depths ranging from 2.8 to 3.5 mm with small internal hyperechoic areas, have poorly defined margins, and show protruding hypoechoic bands extending into the surrounding dermis, occasionally reaching the hypodermis

A total of five dermatofibromas were evaluated in two patients. Clinically, two lesions appeared as flat and three presented as raised pigmented nodules. Four lesions were round and one case was oval in shape. On DG-HFUS dermoscopic imaging every lesion had irregular margins and in two cases a hyperkeratotic and darker center with a delicate pigment network at the periphery (Fig. 4). The other three lesions had a central scar-like white patch typical for dermatofibroma. Upon HFUS, dermatofibromas were located predominantly within the dermis, with depths ranging from 2.4 mm to 3.5 mm, though in 2 cases (Fig. 5.) extension into the hypodermis was observed. The lesions appeared mostly hypoechoic with scattered hyperechoic foci, and were characterized by poorly defined margins. All lesions showed protruding hypoechoic bands extending into the surrounding dermis, reflecting their infiltrative growth pattern (Table 1).

**Table 1:** Clinical and dermoscopy-guided high-frequency ultrasound features of epidermoid cysts and dermatofibromas, highlighting shared characteristics and key differentiating findings. US, ultrasound

Characteristic	Feature	Epidermoid cyst	Dermatofibroma
Lesion shape	Round	4/4	4/5
	Oval	0/4	1/5
Lesion structure	Raised	4/4	3/5
	Flat	0/4	2/5
	Central punctum	2/4	0/5
Lesion colour	Skin coloured	3/4	3/5
	Purple	1/4	0/5
	Dark brown	0/4	2/5
	Central scar-like white patch	0/4	3/5
Margins	Well-defined	4/4	0/5
	Ill-defined	0/4	5/5
Lesion location	Dermis	2/4	3/5
	Dermis-subcutis border	2/4	2/5
US borders	Regular	1/4	0/5
	Irregular	3/4	5/5
Echogenicity	Hypoechogenic	3/4	5/5
	Hyperechogenic	1/4	0/5
Other US signs	Posterior acoustic enhancement	1/4	0/5
	“Submarine sign”	2/4	0/5
	Protruding hypoechoic bands	0/4	5/5

Literature describes dermatofibromas as small (≤ 1 cm), primarily dermal lesions that may extend into the subcutis, with ill-defined, sometimes serrated margins reflecting radial growth [5]. They typically present as heterogeneous hypoechoic nodules, though homogeneous or mixed patterns occur. Additional findings can include punctate calcifications, dermal–epidermal junction thickening, and, less commonly, posterior acoustic enhancement [18]. Study limitations include small sample size and the absence of quantitative analysis and long-term follow-up, limiting generalizability and prognostic interpretation.

DG-HFUS is valuable for evaluating benign cutaneous nodules. Although epidermoid cysts and dermatofibromas may appear clinically similar, they show distinct ultrasound features: epidermoid cysts are well-circumscribed, mixed-echogenic lesions at the dermal–subcutaneous junction, whereas dermatofibromas are smaller, hypoechoic, ill-defined lesions with radiating bands. The combination of dermoscopy and high-frequency ultrasound links surface and subsurface findings, enabling faster, more accurate, and cost-effective diagnosis and supporting lesion differentiation in primary care.

## Data Availability

Data are available if requested.
